# Deep brain stimulation modulates synchrony within spatially and spectrally distinct resting state networks in Parkinson’s disease

**DOI:** 10.1093/brain/aww048

**Published:** 2016-03-26

**Authors:** Ashwini Oswal, Martijn Beudel, Ludvic Zrinzo, Patricia Limousin, Marwan Hariz, Tom Foltynie, Vladimir Litvak, Peter Brown

**Affiliations:** ^1^ Nuffield Department of Clinical Neurosciences, John Radcliffe Hospital, Oxford, UK; ^2^ Medical Research Council Brain Network Dynamics Unit, University of Oxford, UK; ^3^ Wellcome Trust Centre for Neuroimaging, UCL Institute of Neurology, 12 Queen Square, London, UK; ^4^ Unit of Functional Neurosurgery, Sobell Department of Motor Neuroscience and Movement Disorders, UCL Institute of Neurology, Queen Square, London, UK; ^5^ University Medical Centre Groningen, Department of Neurology, University of Groningen, The Netherlands

**Keywords:** deep brain stimulation, Parkinson’s disease, local field potential, resting state networks, magnetoencephalography

## Abstract

Chronic dopamine depletion in Parkinson’s disease leads to progressive motor and cognitive impairment, which is associated with the emergence of characteristic patterns of synchronous oscillatory activity within cortico-basal-ganglia circuits. Deep brain stimulation of the subthalamic nucleus is an effective treatment for Parkinson’s disease, but its influence on synchronous activity in cortico-basal-ganglia loops remains to be fully characterized. Here, we demonstrate that deep brain stimulation selectively suppresses certain spatially and spectrally segregated resting state subthalamic nucleus–cortical networks. To this end we used a validated and novel approach for performing simultaneous recordings of the subthalamic nucleus and cortex using magnetoencephalography (during concurrent subthalamic nucleus deep brain stimulation). Our results highlight that clinically effective subthalamic nucleus deep brain stimulation suppresses synchrony locally within the subthalamic nucleus in the low beta oscillatory range and furthermore that the degree of this suppression correlates with clinical motor improvement. Moreover, deep brain stimulation relatively selectively suppressed synchronization of activity between the subthalamic nucleus and mesial premotor regions, including the supplementary motor areas. These mesial premotor regions were predominantly coupled to the subthalamic nucleus in the high beta frequency range, but the degree of deep brain stimulation-associated suppression in their coupling to the subthalamic nucleus was not found to correlate with motor improvement. Beta band coupling between the subthalamic nucleus and lateral motor areas was not influenced by deep brain stimulation. Motor cortical coupling with subthalamic nucleus predominantly involved driving of the subthalamic nucleus, with those drives in the higher beta frequency band having much shorter net delays to subthalamic nucleus than those in the lower beta band. These observations raise the possibility that cortical connectivity with the subthalamic nucleus in the high and low beta bands may reflect coupling mediated predominantly by the hyperdirect and indirect pathways to subthalamic nucleus, respectively, and that subthalamic nucleus deep brain stimulation predominantly suppresses the former. Yet only the change in strength of local subthalamic nucleus oscillations correlates with the degree of improvement during deep brain stimulation, compatible with the current view that a strengthened hyperdirect pathway is a prerequisite for locally generated beta activity but that it is the severity of the latter that may determine or index motor impairment.

## Teaser

Oswal *et al* . characterise the effect of deep brain stimulation (DBS) on STN–cortical synchronisation in Parkinson–s disease. They propose that cortical driving of the STN in beta frequencies is subdivided anatomically and spectrally, corresponding to the hyperdirect and indirect pathways. DBS predominantly suppresses the former.

## Graphical abstract

**Figure:**
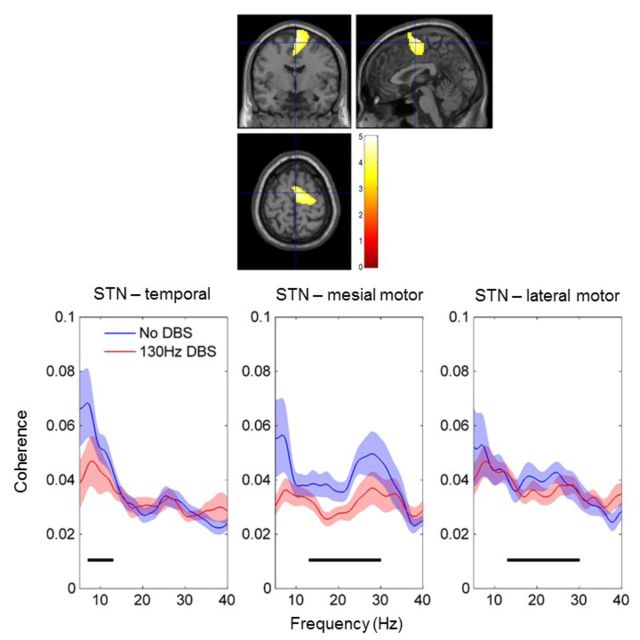
Oswal *et al* . characterise the effect of deep brain stimulation (DBS) on STN–cortical synchronisation in Parkinson–s disease. They propose that cortical driving of the STN in beta frequencies is subdivided anatomically and spectrally, corresponding to the hyperdirect and indirect pathways. DBS predominantly suppresses the former.

## Introduction

Parkinson’s disease is a common disorder, influencing movement and cognition, and is characterized by the progressive degeneration of nigrostriatal dopaminergic neurons. Chronic high frequency deep brain stimulation (DBS) of the subthalamic nucleus (STN) has become an established and effective means of managing the symptoms of Parkinson’s disease, particularly when dopaminergic medications no longer provide consistent benefit or lead to severe dyskinesias. Despite its documented clinical efficacy ( Limousin *et al.* , 1995 ; Deuschl *et al.* , 2006 ; Benabid *et al.* , 2009 ; Schuepbach *et al.* , 2013 ), the precise mechanisms through which DBS exerts therapeutic action remain poorly understood.

Previous studies in rodent and primate models, and in patients with Parkinson’s disease, have implicated excessive oscillatory synchrony, particularly in the beta band (13–30 Hz) as an important pathological feature emerging in the dopamine depleted state. Both the administration of the dopamine precursor levodopa and high frequency DBS of the STN, lead to a suppression of resting beta synchrony ( Brown *et al.* , 2001 ; Eusebio *et al.* , 2011 ; Whitmer *et al.* , 2012 ). Several studies have shown correlations between the extent of beta activity suppression by the dopamine prodrug levodopa and clinical improvements in motor performance ( Kühn *et al.* , 2006 *b* , 2009 ; Weinberger *et al.* , 2006 ; Ray *et al.* , 2008 ).

It has previously been possible to demonstrate the suppression of beta activity locally in the motor cortex ( Whitmer *et al.* , 2012 ), and within the STN during concurrent high frequency DBS using special amplifiers designed to suppress DBS-related artefacts ( Rossi *et al.* , 2008 ; Eusebio *et al.* , 2011 ; Whitmer *et al.* , 2012 ). However, despite advances in our understanding of DBS effects locally within the STN, it is evident from recent work combining either simultaneous EEG or magnetoencephalography (MEG) and intracranial local field potential (LFP) recordings that long range oscillatory synchronization between the STN and cortical regions ( Williams *et al.* , 2002 ; Fogelson *et al.* , 2006 ; Hirschmann *et al.* , 2011 ; Litvak *et al.* , 2011 ), and between the two STNs ( Hohlefeld *et al.* , 2013 ; Little *et al.* , 2013 *b* ), may also be of physiological and pathological relevance in Parkinson’s disease. Such studies have typically used coherence, a linear frequency domain indicator of the similarity between two signals as a measure of functional coupling. Using this approach, two spectrally and spatially distinct STN-cortical resting state networks have been identified in Parkinson’s disease with cortical activity predominantly driving STN activity in both cases ( Hirschmann *et al.* , 2011 ; Litvak *et al.* , 2011 ). The STN is coupled to temporo-parietal areas at alpha band (7–12 Hz) frequencies and to motor/premotor regions at beta band (13–30 Hz) frequencies.

To date no studies have reported what happens to long-range STN–cortical synchronization during concurrent DBS. Here we investigate how DBS influences the synchronization of oscillatory neural activity both locally within the STN, between the STN and cortical regions, and between STNs. We hypothesized that the therapeutic efficacy of DBS relates to both the suppression of local STN beta activity and inter-areal beta band synchronization between STN and motor cortical regions, which drive STN beta activity. In particular, we posited that the cortical drive to the STN through the hyperdirect pathway might be weakened during DBS, given that strengthening of this pathway has been considered a prerequisite for pathological beta activity ( Holgado *et al.* , 2010 ; Moran *et al.* , 2011 ; Marreiros *et al.* , 2012 ).

To test this hypothesis, we studied 15 parkinsonian patients undergoing functional neurosurgery for the insertion of STN electrodes. We performed simultaneous MEG and intracranial LFP recordings and compared differences in STN–cortical coherence topographies at rest and during clinically effective high frequency stimulation of the STN. Our results suggest that beta band synchrony within STN–cortical loops may be subdivided both spectrally and spatially in terms of cortical topography into low and high beta frequency ranges. Although DBS is shown to suppress inter-areal synchrony between STN and motor areas in the high and low beta frequency ranges, it only suppresses local synchronous activity within the STN in the low beta frequency range.

## Materials and methods

### Patient and surgical details

Fifteen patients (mean age 61 years of age; 13 male) who underwent bilateral implantation of STN DBS electrodes were recruited (see Table 1 and Supplementary material for further clinical details).

**Table 1:** Clinical characteristics of patients

Case (postoperative recording day)	Age and gender	Disease duration (years)	Preoperative medication (mg)	UPDRS III Stimulation off/on (OFF medication)							Anatomical location of contacts 0 to 2 on right (R) and left (L) sides
					Total	R A-R	L A-R	R tremor	L tremor	Truncal	
1 (3)	67F	15	LDE 1120	Off	32	6	10	0	0	8	R0 – inside L0 – inside R1 – inside L1 – inside R2 – inside L2 – inside
				On	26	9	10	0	0	3	
2 (6)	67M	7	LDE 2840	Off	43	12	14	0	10	7	R0 – inside L0 – caudal R1 – inside L1 – inside R2 – inside L2 – inside
				On	19	7	6	0	0	3	
3 (5)	67M	8	LDE 200	Off	21	5	6	0	0	6	R0 – inside L0 – inside R1 – inside L1 – inside R2 – inside L2 – inside
				On	17	4	6	0	0	6	
4 (3)	67F	22	LDE 1430	Off	55	11	13	4	4	10	R0 – medial L0 – inside R1 – medial L1 – inside R2 – medial L2 – border
				On	19	4	8	0	1	4	
5 (3)	65M	14	LDE 960	Off	28	3	6	3	3	8	R0 – rostral L0 – inside R1 – medial L1 – inside R2 – medial L2 – inside
				On	11	0	1	2	0	4	
6 (4)	43M	9	LDE 1400	Off	63	17	19	3	2	12	R0 – inside L0 – inside R1 – inside L1 – inside R2 – inside L2 – inside
				On	30	4	13	11	0	1	
7 (3)	61M	8	LDE 400	Off	56	11	11	6	4	12	R0 – rostral L0 – caudal R1 – inside L1 – border R2 – medial L2 – inside
				On	27	5	6	6	6	2	
8 (4)	62M	14	LDE 1640	Off	39	8	17	3	2	5	R0 – caudal L0 – caudal R1 – border L1 – inside R2 – inside L2 – inside
				On	31	8	12	1	2	5	
9 (3)	60M	8	LDE 456	Off	46	12	11	6	3	8	R0 – border L0 – inside R1 – inside L1 – inside R2 – inside L2 – inside
				On	23	4	11	2	1	4	
10 (6)	70M	10	LDE 900	Off	34	8	10	2	2	7	R0 – border L0 – caudal R1 – border L1 – inside R2 – inside L2 – border
				On	34	6	11	2	3	8	
11 (6)	41M	6	LDE 1020	Off	50	14	15	2	2	10	R0 – caudal L0 – inside R1 – border L1 – inside R2 – inside L2 – inside
				On	48	14	18	0	1	11	
12 (6)	58M	12	LDE 1200	Off	38	13	10	0	0	9	R0 – border L0 – inside R1 – inside L1 – inside R2 – inside L2 – inside
				On	13	6	3	1	0	0	
13 (3)	68M	12	LDE 1370	Off	52	8	14	4	5	12	R0 – caudal L0 – inside R1 – border L1 – inside R2 – inside L2 – inside
				On	34	7	4	2	2	9	
14 (3)	60M	12	LDE 1300	Off	33	7	8	7	2	4	R0 – inside L0 – inside R1 – inside L1 – inside R2 – inside L2 – inside
				On	22	3	4	6	1	4	
15 (6)	59M	13	LDE 1900	Off	48	8	10	5	7	9	R0 – inside L0 – inside R1 – inside L1 – inside R2 – inside L2 – inside
				On	27	5	8	1	3	8	

LDE = levodopa dose equivalent. The total UPDRS III motor score and its subscores are presented for the two experimental conditions, no DBS and 130Hz DBS, which are denoted off and on, respectively. A-R denotes the akinesia and rigidity subscore.

### Simultaneous magnetoencephalography and local field potential recordings during DBS

MEG recordings were performed using a CTF 275-channel MEG system (CTF/VSM MedTech). MEG data were sampled at 2400 Hz and stored to disk for subsequent offline analyses.

LFP activity recorded from both STN electrodes was collected at the same time as MEG using a battery-powered and mains optically isolated BrainAmp system (Brain Products). Recordings were performed 3–6 days after electrode implantation, before connection and insertion of the implantable pulse generator ( Table 1 ). Three bipolar channels (0-1, 1-2, 2-3) were recorded from each electrode, so that 0-2 and 1-3 could be derived by the off-line addition of signals from channels 0-1 and 1-2, or 1-2 and 2-3, respectively. LFP recordings were high-pass filtered at 1 Hz in the hardware to avoid amplifier saturation due to large DC offsets. The data were sampled at 2500 Hz, which was the closest available sampling rate to the one used in MEG. LFPs were recorded using a laptop that was optically isolated from the BrainAmp hardware. To fuse the MEG and LFP data with minimal timing distortions we used a synchronization signal (white noise), recorded on both systems (see Oswal *et al.* , 2015 for details).

To permit STN LFP recordings during concurrent stimulation of this nucleus, we used a purpose built stimulation-record amplifier ( Rossi *et al.* , 2007 ; Eusebio *et al.* , 2011 ; Little *et al.* , 2013a ). This allows for monopolar DBS to be administered between either contact 1 or 2 (cathode) of the DBS electrode and an external anodal electrode applied to the patient’s chest wall. By recording bipolar LFP between either contacts 0 and 2 (in the case of contact 1 being the cathode) or between contacts 1 and 3 (in the case of contact 2 being the cathode) of the DBS electrode we were able to exploit the common mode rejection properties of the amplifier. In all our patients, contact pair 0-2 was the one with the greatest resting beta activity and we therefore stimulated between contact 1 and the anode. The stimulation-record amplifier was used in combination with an external DBS stimulator (type 3628, Medtronic Inc.) connected to the intracranial electrode. Right and left STNs were separately stimulated and recorded, while the contralateral unstimulated STN was also recorded using the BrainAmp system.

### Experimental protocol

Recordings were performed after overnight withdrawal from dopaminergic medication. The patient was requested to keep their eyes open and to stay still during recordings. A neurologist was present inside the magnetically shielded scanner room at all times during the recordings, to monitor the wellbeing of the patient and to administer DBS.

The experiment started with a 3 min resting block during which data were collected directly via BrainAmp without the stimulation-record amplifier. The stimulation-record amplifier and external stimulator were then added to the setup and two additional recording runs were performed in which we tested four different stimulation conditions: 0 Hz (no DBS), 5 Hz monopolar DBS, 20 Hz monopolar DBS and 130 Hz monopolar DBS. The BrainAmp system, the stimulation-record amplifier and the external stimulator were kept inside the scanner room during recordings. Two randomly selected stimulation conditions were tested independently for the right and left STNs in each recording run. Each recording run lasted 7 min and included two randomly selected conditions, each lasting 3 min, separated by a 1-min interval for washout of the effect of the first condition. The right STN was stimulated in the first recording run, while the left STN was stimulated in the second recording run.

Monopolar DBS at 130 Hz is widely believed to be the most clinically effective frequency setting ( Benabid *et al.* , 2009 ), but our rationale for including 5 Hz and 20 Hz DBS conditions was to explore whether low frequency DBS, which has previously been shown to exacerbate parkinsonian symptoms ( Chen *et al.* , 2006 *a* ; Eusebio *et al.* , 2008 ), may produce reciprocal clinical and network effects compared to high frequency 130 Hz DBS. In the analysis however, we ended up focusing exclusively on comparing the 0 Hz and the 130 Hz monopolar DBS conditions, since the stimulation artefacts during 5 Hz and 20 Hz DBS at the stimulation frequency and its harmonics were not fully suppressed by the common mode rejection property of our stimulation-record amplifier.

Monopolar, constant voltage DBS, with a stimulation pulse width of 60 µs and an amplitude of 3 V (corresponding to stimulation parameters that are commonly used clinically) was administered by the neurologist. These settings were used in all patients. Although our choice of a fixed stimulation voltage standardized the formal stimulation parameters, variation in tissue impedances may have meant that there was some variability in the current delivered between sides. At the onset of DBS, the stimulation voltage was increased slowly, in increments of 0.5 V, while checking for clinical improvement and for the presence of any stimulation related side effects. Prior to experimentation, clinical assessment of patients confirmed an improvement following DBS. Two patients developed mild and transient lower limb dyskinesias contralateral to stimulation. The clinical response to DBS was formally confirmed using UPDRS Part III rating scores 6 months later during stimulation involving the same parameters.

For further details on data preprocessing and sensor level analyses see the Supplementary material . One of the key challenges of this analysis was the robust handling of artefacts related to DBS, including channel jumps and the effects of stimulation pulses. In related work we have shown that such artefacts can be effectively handled using a combination of jump correction, channel rejection and beamforming such that source analysis is accurately performed ( Oswal *et al.* , 2015 ).

### Analysis of local subthalamic nucleus synchrony

Power spectra of the stimulated STN and the STN contralateral to the stimulated side were computed using the multitaper spectral estimation with a frequency resolution and taper smoothing frequency of 2.5 Hz ( Mitra and Pesaran, 1999 ). Power spectra were normalized, in order to make them comparable across subjects, by dividing each value by the mean power across the 5–40 Hz frequency range in the no DBS condition. The 5–40 Hz frequency range was selected for normalization since the bandpass range of our stimulation-record amplifier was 4–40 Hz. Coherence between STNs was also computed using multitaper spectral estimation with a frequency resolution and taper smoothing frequency of 2.5 Hz. To test for the effects of DBS on coherence or power, the spectral time series were converted to 1D images and smoothed with a 2.5 Hz Gaussian kernel to ensure conformance to the assumptions of random field theory. The images were then subjected to a paired *t* -test in SPM to determine regions in frequency space where DBS-associated spectral changes were significant.

In addition to modelling subject-specific dependencies in the recordings from the two hemispheres, we included side as an additional categorical variable for each subject to account for potential differences between the recordings from the right and left STNs. A covariate representing each patient’s preoperative levodopa equivalent dose ( Table 1 ) was also introduced to account for differences in medication doses, in addition to a covariate representing the contralateral limb akinesia-rigidity score, which served to account for differences in disease severity and lateralization. Findings were similar if this last covariate was omitted (data not shown). This was checked lest the covariate of OFF drug contralateral limb akinesia score served to regress out effects due to stimulation, which might occur if stimulation effects were correlated with the OFF drug scores. All analyses were corrected for multiple comparisons using random field theory and reported findings are significant with familywise error (FWE) correction at the cluster level ( *P* < 0.05 corrected, cluster forming threshold *P* < 0.01 uncorrected).

### Determining the spatial and spectral profile of coherence changes induced by DBS

Brain areas coherent with the STN LFP were localized using dynamic imaging of coherent sources (DICS) beamforming ( Gross *et al.* , 2001 ) as described in our previous publications ( Litvak *et al.* , 2010 , 2012 ; Oswal *et al.* , 2013 , 2014 ). Beamforming rests on a linear projection of sensor data using a spatial filter that is computed from the lead-field of a location of interest and either the data covariance or the cross-spectral density matrix ( Van Veen *et al.* , 1997 ; Gross *et al.* , 2001 ). Lead-fields were computed using a single shell head model ( Nolte, 2003 ). The model was generated in Statistical Parametric Mapping (SPM) based on the patient’s preoperative structural MRI and fiducial-based co-registration was performed as described in Litvak *et al.* (2010) . A beamformer regularization of 0.01% was used as per Litvak *et al.* (2010) .

The source space was defined as a 5 mm spaced grid limited to the inner skull compartment. The goal of this particular analysis was to generate a 3D image showing coherence between the STN and all brain regions during the DBS and the no DBS conditions. Given previous studies highlighting the existence of separate alpha (7–12 Hz) and beta (13–30 Hz) band resting STN–cortical networks, we initially restricted our DICS beamformer analysis to these two bands ( Hirschmann *et al.* , 2011 ; Litvak *et al.* , 2011 ; Oswal *et al.* , 2013 ) ( Supplementary material ).

For each subject and each hemisphere, coherence images were generated for the two frequency bands in both experimental conditions. Half of the resulting images (all left STN images) were reflected across the median sagittal plane to allow comparison of ipsilateral and contralateral sources to the STN regardless of original STN side. These images were then subjected to a 2 × 2 factorial ANOVA, with frequency (alpha versus beta) and stimulation (no DBS versus 130 Hz DBS) as factors in SPM. Subject, side, medication dose and contralateral limb akinesia-rigidity were also included as covariates, as described above. We constructed regions of interest separately for the alpha and beta networks by performing a two sample *t* -test of alpha and beta band DICS beamformer images and testing for regions where beta band coupling was significantly greater than alpha band coupling and vice versa. For the definition of these regions of interest we used independent data from the 3-min rest block. All analyses were corrected for multiple comparisons using random field theory and reported findings are significant with familywise error (FWE) correction at the cluster level ( *P* < 0.05 corrected, cluster forming threshold *P <* 0.01 uncorrected). DICS beamformer images for cortical power were also generated and analysed in a similar way.

The direction of the simple main effect of stimulation was estimated by performing one-tailed *t* -tests. For example, for the beta band we could determine brain regions where coherence was significantly decreased by DBS or vice versa. Similarly *t* -contrasts could be specified to determine the directionality of any interaction between the two factors. All analyses were corrected for multiple comparisons as explained above. Peak voxels resulting from group-level SPMs of the simple main effects underwent further analysis for coherence and clinical correlations.

First, we performed extraction of the time series data for each hemisphere from the location of the peak. We also extracted source time series from a location within the boundaries of primary motor cortex (M1), positioned at MNI co-ordinates at 37 −18 53 (right M1) and −37 −18 53 (left M1) ( Mayka *et al.* , 2006 ; Litvak *et al.* , 2012 ) inverse-normalized to account for the individual head size ( Supplementary material ).

Once individual trial time series were extracted for the different experimental conditions, coherence was computed between the reconstructed source and the reference channel using multitaper spectral estimation with a frequency resolution and taper smoothing frequency of 2.5 Hz ( Mitra and Pesaran, 1999 ). To provide additional immunity from artefacts, robust averaging was applied to both the cross-spectral density estimates and the auto-spectra across trials prior to computing coherence ( Wager *et al.* , 2005 ; Litvak *et al.* , 2012 ; Oswal *et al.* , 2015 ).

Three dimensional images of cortical power were also computed using the DICS beamforming approach. Subsequently power estimates for the alpha and beta bands were compared in the no DBS and 130 Hz DBS conditions using paired *t* -tests.

### Directionality of subthalamic nucleus–cortical coupling

To determine the effective directionality of functional coupling between the cortex and the STN we computed a non-parametric variant of spectral Granger causality ( Brovelli *et al.* , 2004 ). In comparison to standard spectral domain Granger causality based approaches, this does not rely on fitting a multivariate autoregressive model and hence makes fewer assumptions about the data ( Dhamala *et al.* , 2008 ). To determine the significance of coupling, we compared the Granger estimate of original data to that of surrogate time reversed data using a paired *t* -test across hemispheres ( Haufe *et al.* , 2013 ). For further details and for details of our approach for estimating phase delays, see the Supplementary material .

### Correlation with clinical features

The aim of this analysis was to determine whether changes in motor function induced by DBS correlated with associated electrophysiological changes within STN–cortical circuits. Limb akinesia-rigidity was assessed using the Unified Parkinson’s Disease Rating Scale (UPDRS) part III (items 22 to 26) ( Fahn and Elton, 1987 ). Scoring was performed by an experienced neurologist after patients had been withdrawn from their medication overnight and were in an OFF state. To assess the effects of DBS, contralateral limb akinesia-rigidity was assessed OFF drug immediately prior to surgery, and ∼6 months after the implantation of the internal pulse generator with DBS at the same settings and contacts as used in the MEG experiment. Pearson’s correlation was used to correlate changes in contralateral limb akinesia-rigidity with DBS (i.e. the change in akinesia-rigidity scores between OFF drug, before DBS, and those OFF drug, on DBS made after implantation) to changes in LFP power and LFP-MEG coherence during DBS performed OFF drug, 3–6 days after electrode implantation. UPDRS assessments were not performed at the time of the MEG recordings. We only included coherence with MEG signals that showed statistically significant modulation by DBS at the group level. Note that only changes in contralateral akinesia-rigidity scores were used in correlations as it is these that have previously been reported to best correlate with beta activity in cortico-basal ganglia circuits ( Kühn *et al.* , 2006a , 2009 ; Ray *et al.* , 2008 ; Özkurt *et al.* , 2011 ; Sharott *et al.* , 2014 ). All correlations were corrected for multiple comparisons using a Bonferroni correction with an alpha of 0.05.

## Results

### DBS effects on local power

#### DBS specifically suppresses 11–14 Hz activity in the subthalamic nucleus

**Figure 1:**
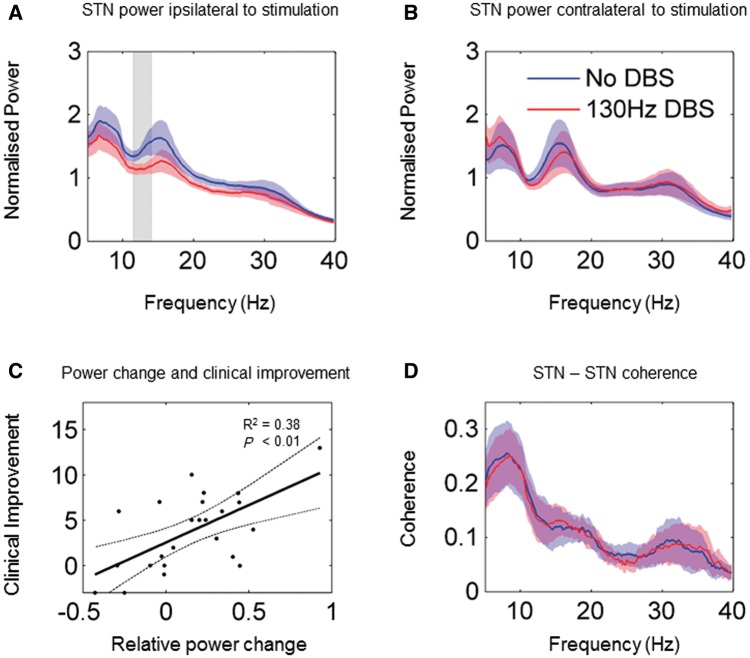
**Changes at STN level.** ( **A** ) Group average normalized power spectra of the stimulated STN during the no DBS and the 130 Hz DBS conditions. The shaded regions represent standard errors of the mean. In the no DBS condition spectral peaks are evident in the alpha (8–12 Hz), low beta (13–21 Hz) and high beta (21–30 Hz) frequency ranges. DBS results in a suppression of power in the frequency range indicated by the grey bar (11–14 Hz). ( **B** ) Group average spectra of the unstimulated STN contralateral to the stimulated side. There are no significant spectral changes associated with DBS. ( **C** ) Correlation between DBS-related changes in relative power in the 11–14 Hz frequency range across hemispheres and changes in contralateral hemibody akinesia/rigidity scores. A linear regression line is plotted with 95% confidence intervals indicated by the dotted lines. Clinical improvement (no DBS-130 Hz DBS contralateral rigidity/akinesia scores) correlated significantly with suppression of 11-14 Hz power (presented as No DBS-130 Hz DBS; F = 13.6, r ^2^ = 0.38, *P =* 0.0013). ( **D** ) Group average coherence spectra between stimulated and contralateral STNs. Unilateral DBS does not affect coherence between the two STNs.

Figure 1 shows data averaged from 24 subthalamic electrodes. Figure 1 A and B demonstrate the mean spectral power profiles recorded across bipolar contacts 0-2 of the stimulated STN electrode and of the STN electrode contralateral to the stimulated side during the two experimental conditions. At rest (no DBS condition), there are spectral peaks centred at ∼7 Hz, ∼15 Hz and ∼30 Hz. These approximately correspond to the alpha, low beta and high beta frequency bands, respectively, although these bands have been previously defined according to cortical reactivity and do not precisely match with subcortical spectral reactivities. DBS resulted in a significant suppression of activity in the stimulated STN over 11–14 Hz, which for simplicity we will consider within the low beta frequency band. The 11–14 Hz range is highlighted in the grey in Fig. 1 A [peak *t* (31) = 2.9, FWE *P* < 0.05]. This effect was lateralized, with no significant change in power in the contralateral, unstimulated STN [ Fig. 1 B, peak *t* (31) = 0.9, FWE *P* > 0.2].

In contrast to the STN, cortical power in the alpha and beta frequency ranges was not significantly influenced by DBS following correction for multiple comparisons using random field theory ( *P <* 0.05 corrected, cluster forming threshold *P <* 0.01 uncorrected). Supplementary Fig. 3 shows images of *t* -statistics for the comparison of cortical power on and off DBS, separately for the alpha and beta bands.

#### Change in subthalamic nucleus local field potential 11–14 Hz activity correlates with DBS efficacy

Next we investigated whether clinical improvements in motor scores associated with DBS correlated with DBS-associated changes in power in the STN. To optimize our chance of picking up any correlation we separately averaged power for both experimental conditions over the precise frequency range (11–14 Hz) showing significant power modulation, and then subtracted these values for each side. We found a significant positive correlation between DBS related power suppression in the 11–14 Hz band in the STN and improvements in contralateral hemibody akinesia-rigidity scores (F = 13.6, r ^2^ = 0.38, *P* = 0.0013).

### DBS selectively suppresses coupling between subthalamic nucleus and a mesial motor beta band network in Parkinson’s disease

**Figure 2:**
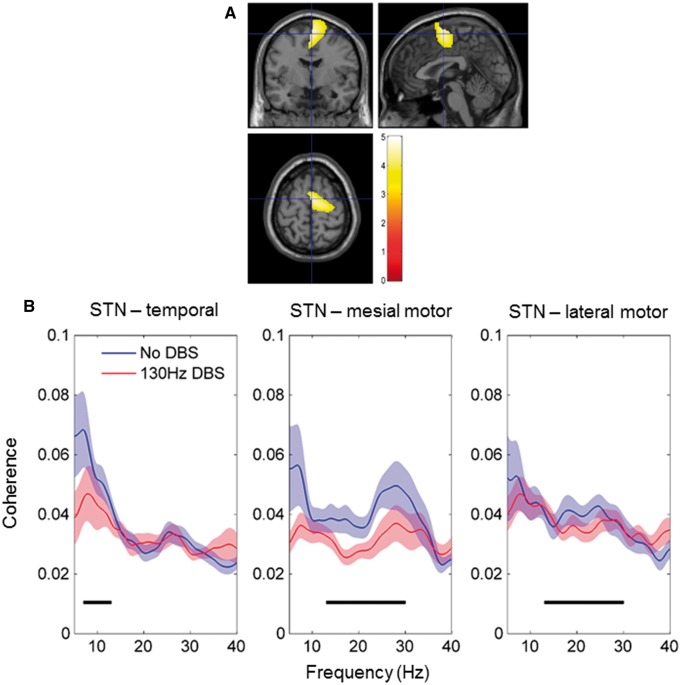
**Effect of 130 Hz DBS on STN-MEG coherence.** ( **A** ) Group thresholded SPM of the simple main effect of DBS on the beta band network. The coloured region indicates areas where DBS significantly suppressed STN-cortical beta band coherence. Values indicated by the colour bar are *t* -statistics. The SPM is superimposed on a T _1_ -weighted canonical MRI with cross-hairs centred on the value of the peak t statistic at MNI co-ordinates 2 −4 64. For the simple main effect of DBS on the alpha band network, no clusters survived correction for multiple comparisons. ( **B** ) Group mean spectra of coherence computed between the STN and locations of the peak t statistic of simple main effects separately for the alpha and beta networks. Black bars denote alpha (8–12 Hz) and beta bands (13–30 Hz) in the left and right hand side plots, respectively.

There was no significant modulation of the coherence between the two STNs during DBS [peak *t* (31) = 0.46, FWE *P* > 0.9]. Comparison of subcortico-cortical coherence off and on DBS revealed a simple main effect of stimulation on coherence in the beta band such that broad beta band coherence was suppressed in relatively focal mesial pre-motor regions, indicated in Fig. 2 A. The coloured region indicates voxels that survived cluster level correction as described in the ‘Materials and methods’ section. The location of the peak *t* -statistic [peak *t* (77) = 6.1, FWE *P <* 0.01] corresponds to MNI coordinates 2 −4 64 and the region encompasses mesial pre-motor areas such as the supplementary motor area (SMA), but not the pre-SMA. In contrast there was no simple main effect of stimulation on coherence in the alpha band [peak *t* (77) = 3.0, FWE *P* > 0.1]. Coherence images averaged across subjects, for the alpha and beta bands, on and off DBS are shown in Supplementary Fig. 1 C and D.

Source extracted coherence spectra are shown in Fig. 2 B. Source time series were extracted from a region within the primary motor cortex (M1), which we will refer to as a lateral motor area, and from the locations of the peak *t* -statistic of the simple main effect of stimulation for each frequency band. In the alpha band coherence peaked in the superior temporal gyrus and coherence spectra were similar in the no DBS and the 130 Hz DBS conditions ( Fig. 2 B). In contrast, in the beta band where coherence peaked in mesial premotor areas of the cortex, DBS resulted in a suppression of coupling between the STN and these regions. Crucially, however, coupling between STN and lateral motor areas was not influenced by DBS.

### Ratio of low and high beta band coupling differs between the STN-mesial premotor and STN-lateral motor networks

**Figure 3:**
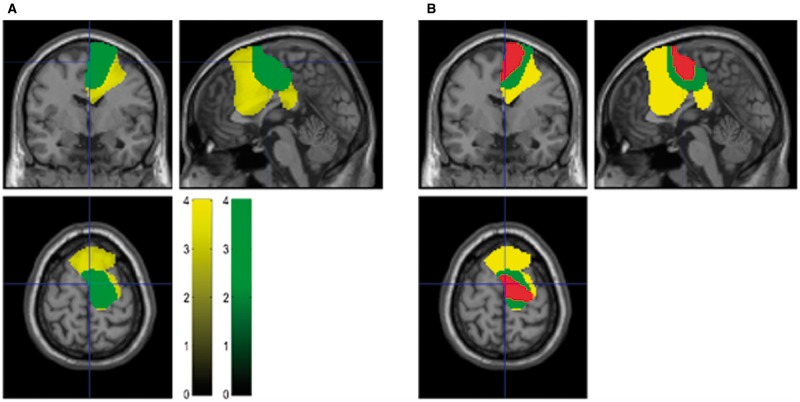
**Differences between STN-MEG coherence in low and high beta bands.** ( **A** ) Thresholded SPMs superimposed onto a T _1_ -weighted canonical MRI with region in yellow representing voxels where STN-cortical coupling was significantly greater in the beta band than in the alpha band in the resting block. Region in green represents voxels within the resting beta network where STN-cortical coupling in the high beta frequency range (21–30 Hz) exceeded coupling within the low beta range (13–21 Hz). Colour bars for the yellow and green images represent t-statistics. ( **B** ) Resting beta network (yellow), high beta network (green) and the cortical region where STN-cortical beta band coupling is suppressed by DBS (red) are all superimposed.

At rest, in the no DBS condition, STN–cortical coherence in the high beta range appeared to dominate over that in the low beta range in the STN-mesial premotor network ( Fig. 2 B). Given this, we explored whether the topography of STN-cortical coupling in the broad beta band could be topographically further refined in terms of any relative dominance of low or high beta frequencies. Accordingly, we computed DICS beamformer images as before for data from the no DBS condition, but subdivided the beta band into a low (13–21 Hz) and high (21–30 Hz) ranges. A two sample *t* -test was then used to determine regions within the resting state beta network where STN-cortical coupling was greater in the high beta band than in the low beta band and vice versa. High beta band coupling was significantly greater than low beta band coupling in posterior mesial pre-motor regions encompassing SMA [ Fig. 3 A, peak *t* (81) = 5.9, *P <* 0.01]. However, the reverse contrast, to test for regions where low beta coupling was greater than high beta coupling was not significant [peak *t* (81) = 3.3, *P* > 0.2]. Thus in posterior mesial pre-motor areas, such as SMA, STN–cortical cortical coherence dominated in the high as opposed to the low beta frequency range. Outside of this mesial region, there was no frequency preference of STN-cortical beta band coupling.

The region where resting STN-cortical coupling was greater in the high beta band than in the low beta band largely coincided with that in which beta band STN-cortical coupling was suppressed by DBS. This is highlighted in Fig. 3 B, where the green areas represent the regions where high beta band coherence dominates and the area in red represents regions where DBS suppressed STN-cortical beta band coupling.

An important question arising from the above findings is whether DBS predominantly therefore suppresses high, rather than low, beta STN-cortical coherence. To address this, we constructed a 2 × 2 × 2 ANOVA repeated measures with factors band (low versus high beta), cortical topography (mesial versus remaining motor) and stimulation (no DBS versus 130 Hz DBS). Mesial motor areas were defined as all voxels incorporated within the region where high beta STN–cortical coupling was greater than low beta STN–cortical coupling ( Fig. 3 A and B). The remaining motor region was defined as all of the voxels outside the mesial region, but inside the resting beta band network ( Fig. 3 A and B). Coherence values of voxels within these regions were averaged to yield a single value for each level of the ANOVA. Mean coherence values across subjects are shown in Supplementary Table 1 . As expected there was a main effect of stimulation, with DBS producing significant beta suppression [ *F* (1,182) = 9.9, *P <* 0.01], but no main effect of cortical topography [ *F* (1,182) = 0.82, *P* = 0.37] or band [ *F* (1,182) = 0.06, *P* = 0.81]. There were, however, interactions between cortical topography and band [ *F* (1,182) = 4.2, *P <* 0.05] and between stimulation and cortical topography [ *F* (1,182) = 11.1, *P <* 0.01]—such that high beta band coupling was greater in mesial rather than in remaining motor regions and DBS predominantly reduced beta activity in the mesial areas. Crucially however there was no interaction between stimulation and band [ *F* (1,182) = 2.0, *P* = 0.16], nor was there an interaction between all three factors [ *F* (1,182) = 0.98, *P =* 0.32]. The lack of an interaction between stimulation and band was corroborated by considering the effect of DBS on coherence with 1 Hz resolution. This reinforced that the suppression of coherence by DBS was fairly evenly distributed across the broad beta band ( Fig. 2 B).

### Phase differences differ between low and high beta band networks linking motor cortex and subthalamic nucleus

**Figure 4:**
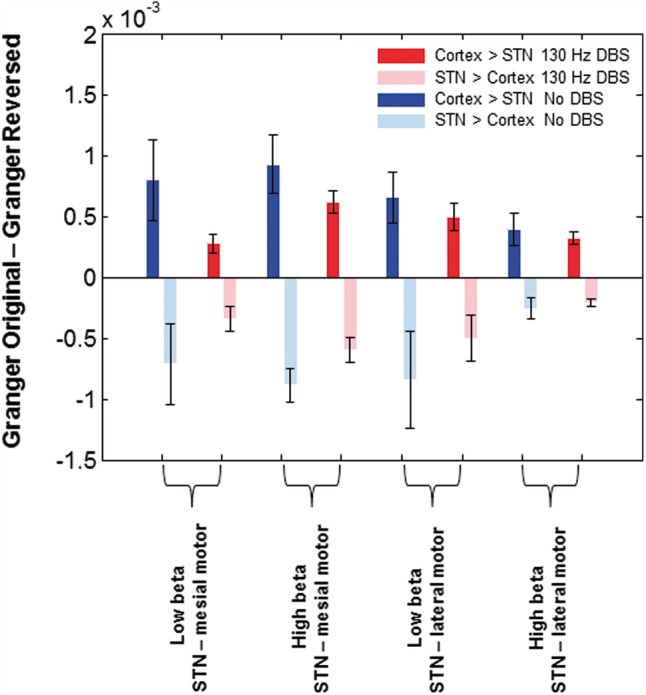
**Results of Granger causality analysis.** Group mean difference in Granger causality between original and time reversed data in no DBS and 130 Hz DBS conditions for the high and low beta sub-bands. Source time series were extracted from the location of the peak *t* -statistic of the simple main effect of DBS in the beta band (at MNI co-ordinates 2 −4 64 corresponding to mesial motor regions) and additionally from a source within primary motor cortex (M1), which we term lateral motor. The difference in Granger causality is significantly greater than zero in the direction of cortex leading the STN for both cortical regions in the no DBS and the 130 Hz DBS conditions (see ‘Results’ section).Vertical bars represent standard errors of the mean. In contrast the difference in Granger causality is less than zero in the direction of STN leading the cortex, confirming that cortical activity led that in STN.

The evidence above suggests that mesial premotor cortex involves more high than low frequency beta coupling, and that DBS of the STN preferentially suppresses coupling with these cortical areas, but without distinguishing between the lower and upper beta band frequency ranges. Next we sought to further characterize couplings in the lower and upper beta band and to test the hypothesis that they might relate to indirect and hyperdirect connectivity with the STN, respectively. First, we confirmed that coupling involved cortical driving of STN, both for the upper and lower beta bands during and without STN DBS. Figure 4 plots the mean difference in upper and lower beta band Granger causality between the original and time reversed data, averaged across hemispheres for the no DBS and 130 Hz DBS conditions. For this analysis we used source time series extracted from the location of the peak *t* -statistic of the simple main effect of DBS in the beta band (at MNI coordinates 2 −4 64, corresponding to mesial premotor regions) and from a source within the primary motor cortex (lateral motor source at MNI coordinates 37 −18 53). The difference in Granger causality was significantly greater than zero in the direction of cortex leading the STN for both the no DBS and the 130 Hz DBS conditions, for both cortical regions and both beta sub-bands. For the STN–mesial network, one sample *t* -test statistics for the low beta sub-band were: *t* (23) = 2.1, *P =* 0.02 in the no DBS condition, and *t* (23) = 1.9, *P =* 0.03 in the 130 Hz DBS condition. For the same location and the high beta sub-band one sample *t* -tests were: *t* (23) = 2.6, *P <* 0.01 in the no DBS condition, and *t* (23) = 2.5, *P =* 0.01 in the 130 Hz DBS condition. Similarly for the STN-lateral motor network and the low beta sub-band one sample *t* -test statistics for the low beta sub-band were: *t* (23) = 2.2, *P =* 0.02 in the no DBS condition, and *t* (23) = 2.1, *P =* 0.02 in the 130 Hz DBS condition. For the high beta sub-band of the STN-M1 network one sample *t* -test statistics for the low beta sub-band were: *t* (23) = 2, *P =* 0.03 in the no DBS condition, and *t* (23) = 1.9, *P =* 0.03 in the 130 Hz DBS condition. To investigate the dependence of cortical Granger causal drive on the beta sub-band, DBS state and cortical location we constructed a 2 × 2 × 2 ANOVA with factors sub-band (low versus high beta), DBS (no DBS versus DBS) and cortical location (mesial premotor versus lateral motor). The only significant finding was an interaction between DBS and cortical location [ *F* (1,182) = 4.4, *P <* 0.05] such that Granger causal drive was reduced by DBS, but in mesial rather than in lateral motor areas.

**Figure 5:**
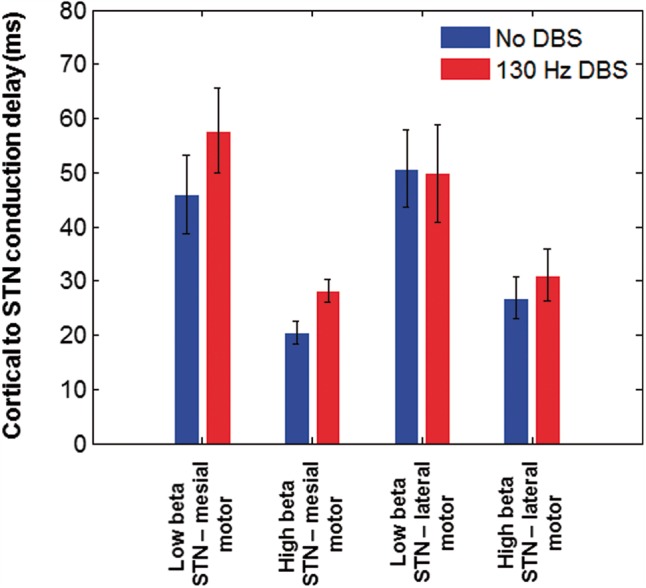
**Estimated conduction delays.** Group mean conduction delays between cortical regions (Source within M1and the location of the peak t-statistic for the simple main effect of DBS on the beta network at MNI co-ordinates 2 −4 64) and the STN are shown for the low (13–21 Hz) and high beta (21–30 Hz) sub-bands in both experimental conditions. The vertical bars represent standard errors of the mean. Conduction delays are significantly greater for the low beta frequency range than for the high beta frequency range.

Second, we sought to estimate the net time delays between the cortex and STN. There was a significant linear relationship between phase and frequency allowing net delays to be estimated in 18 of 24 hemispheres ( Fig. 5 ). For the no DBS condition, the mean delays between the location of the peak *t* -statistic of the simple main effect of DBS in the beta band in the mesial premotor cortex and the STN was 46 ms and 20 ms for the low and high beta frequency ranges, respectively ( Fig. 5 ). To explore the dependence of delays on the beta sub-band, DBS state and cortical location we constructed a 2 × 2 × 2 ANOVA with factors sub-band (low versus high beta), DBS (no DBS versus DBS) and cortical location (mesial premotor versus lateral motor). There was a main effect of sub-band such that net delays were lower for the high beta sub-band [ *F* (1,84) = 16.85, *P <* 0.01], but no other significant main effects or interactions.

### DBS effects on resting state networks: lack of correlation with DBS efficacy

Finally, we investigated whether clinical improvements in motor scores associated with DBS correlated with DBS-associated changes in STN-cortical beta band coherence across subjects. We used coherence values averaged over the broad 13–30 Hz beta range and also separately across the 13–21 Hz and 21–30 Hz sub-bands. None of the comparisons yielded significant correlations.

## Discussion

**Figure 6:**
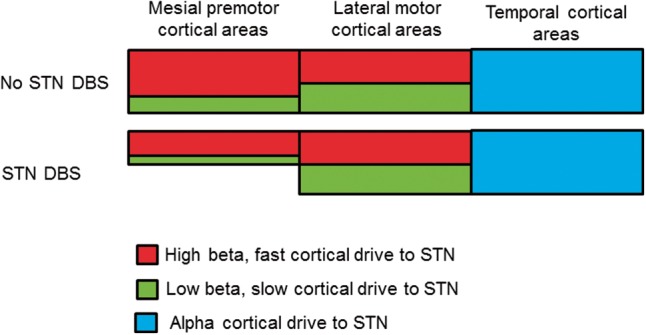
**Schematic of DBS effects on STN-cortical networks.** Mesial premotor areas, including the SMA are preferentially coupled to the STN at rest at high (red) rather than at low (green) beta frequencies. This pattern is not observed for lateral motor areas, including parts of M1, where coupling to the STN is more evenly distributed across beta frequencies. STN DBS acts to suppress driving of the STN by mesial premotor regions in the low and high beta sub-bands. Crucially these effects of DBS are limited to the STN-mesial premotor network and are not observed in the patterns of DBS reactivity of the STN-lateral motor network (see ‘Discussion’ section).

We studied the effects of STN DBS on synchronized oscillatory activity within and between the STN and cortical structures in patients with Parkinson’s disease. We found that DBS had contrasting effects on local activity in the STN and on the coupling of this to different distal sites. DBS suppressed synchronized neuronal activity within the STN, preferentially at low beta rather than high beta frequencies. Suppression in the low beta band correlated with motor improvement. In contrast, DBS suppressed the coupling of STN to cortical motor regions across the entire beta frequency band, and this did not correlate with clinical improvement. The effect of STN DBS on coupling with cortex was spatially selective and restricted to mesial premotor cortical areas. The latter were distinguished from more lateral motor cortical areas, including parts of the primary motor cortex, by coupling with STN that was stronger in the high than in the low beta frequency band, whereas these were more evenly balanced over lateral motor cortical areas. Coupling between STN and cortical motor areas consisted of a predominant drive from cortex to STN, but coupling in the higher beta frequency band had a much more rapid effect on STN activity than that in the lower beta frequency band. These observations raise the possibility that cortical connectivity with the STN in the high and low beta bands may reflect coupling mediated predominantly by the hyperdirect and indirect pathways to STN, respectively ( Alexander and Crutcher, 1990 ; DeLong, 1990 ). Our findings also imply that the hyperdirect connectivity, characterized by activity in the high beta band, relatively dominates in mesial premotor cortical areas. It was the coupling between these latter areas and STN that was most sensitive to DBS (see summary schema in Fig. 6 ). Collectively our results are consistent with the hypothesis that the therapeutic effects of STN DBS are mediated predominantly by suppression of low beta activity locally within the STN ( Eusebio *et al.* , 2012 ). In addition, the present results suggest that DBS may attenuate the mesial cortical drive supported by the hyperdirect pathway. This potentially is important, as the strengthening of the hyperdirect pathway drive has been proposed to be a necessary prerequisite for the development of exaggerated beta activity in the STN ( Holgado *et al.* , 2010 ).

### Study limitations

Before considering the functional and clinical significance of our findings it is prudent to highlight the limitations of the study. Major limitations were 4-fold, with each possibly serving to underestimate the scale of either coupling at rest, changes in coupling with DBS or correlations between clinical scores and electrophysiological variables. First, as recordings were made a few days after electrode implantation they are likely to be affected by a postoperative stun effect, which has been associated with a reduction in spontaneous beta activity in the STN and temporary amelioration of parkinsonism ( Chen *et al.* , 2006b ). Second, the timings of our experimental conditions were determined by clinical constraints, particularly patient fatigue, in this postoperative period. Specifically, the wash-out period between conditions was only 1 min so that effects persisting beyond cessation of stimulation may have tended to obscure differences between stimulation regimens. Likewise, signals were analysed that were recorded as little as 20 s (mean 110 s) after the onset of 130 Hz DBS, thereby likely failing to reflect the full effect of stimulation ( Lopiano *et al.* , 2003 ; Temperli *et al.* , 2003 ). However, even 20 s of stimulation is sufficient to have some effects on beta activity ( Wingeier *et al.* , 2006 ), and even after 200 s of STN DBS beta power changes have resolved within 30 s of stimulation offset ( Kühn *et al.* , 2008 ). Still, the time course of wash-out effects with respect to subcortico-cortical coherence is less clear, but reassuringly we did not observe any significant differences in split half coherence analysis of each recording condition ( Supplementary material ). Third, we correlated the difference in UPDRS III OFF drug motor scores that were collected prior to surgery and ∼6 months following surgery with electrophysiological parameters that were recorded within a few days following surgery. This difference in timing may serve to reduce sensitivity to detect correlation, making our analyses more conservative. Hence the correlation between DBS induced low beta power suppression in the STN and improvements in hemibody UPDRS motor scores may have been under-estimated and a weak correlation between STN-cortical beta coherence suppression and hemibody UPDRS motor score improvements missed. However, it is likely that the relative contrast between these two correlations would remain. Finally, we focussed on linear interactions between sites, in the form of coherence, and this will have missed non-linear phenomena such as cross-frequency phase-amplitude coupling or stochastic, non-oscillatory synchronization between the cortex and STN ( Özkurt *et al.* , 2011 ; de Hemptinne *et al.* , 2015 ).

### DBS influences spatially, functionally and spectrally distinct cortico- subthalamic nucleus networks

Previous resting state recordings in patients with Parkinson’s disease have demonstrated the existence of spatially and spectrally segregated STN–cortical networks. The STN is coupled to motor regions in the beta band, and also to temporal regions in the alpha frequency range. We replicated these findings in our study, and report novel differential influences of DBS on activity within these networks.

First, DBS did not influence coupling between the STN and temporal areas in the alpha band. While this is in keeping with a lack of the effect of the dopamine prodrug levodopa on this network at rest, it is worth noting that movement-related suppression of STN-temporal alpha band coupling is enhanced by levodopa and that this change correlates with clinical motor impairments ( Oswal *et al.* , 2013 ). The precise role of the alpha band STN-temporal network remains to be fully elucidated, but it may reflect activity in the anatomical connection between the STN and superior temporal gyrus identified in human tractography studies ( Brunenberg *et al.* , 2012 ).

In contrast, DBS suppressed beta band synchronization between the STN and cortical motor regions. However, here our results suggested that this STN-cortical motor circuit can be further divided on functional grounds, including reactivity to DBS. Thus STN DBS suppressed beta band synchronization between the STN and mesial motor regions encompassing the SMA, but had no effect on the coupling between the STN and lateral motor regions that included parts of primary motor cortex. This differential reactivity to DBS was paralleled by spectral differences between these motor sub-circuits. Those mesial areas that uncoupled from the STN during DBS were more strongly coupled to the STN in the high beta than the low beta frequency range. In contrast, motor areas outside of this mesial premotor region exhibited beta band coupling with the STN that was more balanced between low and high frequencies within the beta band. A similar dominance of STN coherence with mesial cortical areas at higher beta frequencies has also been inferred from EEG studies, although without the spatial resolution possible here ( Fogelson *et al.* , 2006 ). Note that both mesial and lateral motor regions in the cerebral cortex were Granger causal for activity in the STN in the beta band—an important observation that rules out direct pick-up of cortical activities by our DBS electrodes.

Does this functional division of STN coupling with mesial and lateral motor regions find any correlate in anatomical and functional imaging studies? Tractography studies in both healthy subjects and in patients with Parkinson’s disease have demonstrated the existence of anatomical connectivity between the SMA and the STN consistent with the hyperdirect pathway identified in rodents and non-human primates ( Baudrexel *et al.* , 2011 ; Brunenberg *et al.* , 2012 ; Lambert *et al.* , 2012 ). Furthermore, PET studies demonstrate STN DBS predominantly reduces regional cerebral blood flow to mesial premotor regions including the SMA ( Hershey *et al.* , 2003 ; Asanuma *et al.* , 2006 ; Karimi *et al.* , 2008 ). In line with the proposal that the hyperdirect pathway characterized by activity in the upper beta frequency band might dominate the drive from mesial motor regions to STN a study placing electrocorticographic electrodes over mesial motor cortical areas confirmed high beta band coherence between these electrodes and STN at rest, although coherence changes were not reported during DBS ( Whitmer *et al.* , 2012 ). Conversely, another recent study placed electrocorticographic electrodes directly over the primary motor cortex and reported peak coherence in the low beta frequency band ( de Hemptinne *et al.* , 2015 ).

We hypothesize that STN coherence with cortical motor regions in the high and low beta frequency ranges might correspond to activities predominantly driven by the classical hyperdirect and the indirect pathways to the STN, respectively ( Alexander and Crutcher, 1990 ; DeLong, 1990 ). These pathways overlap in their cortical topography, although the present results suggest that the hyperdirect pathway tends to dominate in mesial motor areas ( Supplementary Table 1 ). In line with this, Granger causality was consistent with cortical driving of STN in both sub-bands and the phase delays to STN were much shorter for the high beta than the low beta frequency range. Indeed, the differential net temporal lead of cortex in the two frequency sub-bands compares very favourably with that reported in an independent patient cohort studied with EEG ( Fogelson *et al.* , 2006 ). The relatively long (20 ms) delay to STN in the high beta band may arise because activities in the hyperdirect and indirect pathways are only relatively and not absolutely spectrally distinct (i.e. there may be partial mixing). In this case estimates of the delay in the hyperdirect pathway will be exaggerated by the indirect component overlapping in frequency ( Cassidy and Brown, 2003 ; See Supplementary material and Supplementary Fig. 2 for simulation). Note, however, that mixing can only act to negate differences in phase delays between circuits, and substantial differences between phase delays from cortex to STN remained in the upper and lower beta frequency bands despite potential mixing.

Numerous prior studies have proposed excessive hyperdirect pathway activity as an important factor in the motor dysfunction in Parkinson’s disease, with some of these providing experimental or modelling evidence that DBS selectively suppresses this increased hyperdirect pathway coupling ( Leblois *et al.* , 2006 ; Dejean *et al.* , 2008 ; Gradinaru *et al.* , 2009 ; Holgado *et al.* , 2010 ; Baudrexel *et al.* , 2011 ; Tachibana *et al.* , 2011 ; Moran *et al.* , 2011 ; Marreiros *et al.* , 2012 ; Kang and Lowery, 2013 , 2014 ; Kahan *et al.* , 2014 ). The lack of correlation between the weakening of STN coherence with mesial cortical motor areas and clinical improvement upon DBS would be consistent with the view that a strengthened hyperdirect pathway is a prerequisite for beta activity, in so far as this reflects motor dysfunction, but not sufficient ( Holgado *et al.* , 2010 ; Moran *et al.* , 2011 ; Marreiros *et al.* , 2012 ).

Observations in rodents suggest that the effects of DBS on the hyperdirect pathway may be secondary to the antidromic activation of corresponding axons ( Dejean *et al.* , 2008 ; Gradinaru *et al.* , 2009 ; Li *et al.* , 2012 ). This is supported in patient studies by recordings of STN DBS evoked cortical potentials of short latency that are able to follow high stimulation frequencies ( Ashby *et al.* , 2001 ; Devergnas and Wichmann, 2011 ; Walker *et al.* , 2012 ). These evoked potentials are reported to be maximal in amplitude over mesial cortical areas, despite the presence of more lateral burr holes ( Ashby *et al.* , 2001 ; Walker *et al.* , 2012 ). In line with this, the present results suggest that the hyperdirect pathway may be disproportionately represented in mesial cortical motor areas. It remains to be determined why DBS in the STN might preferentially affect inputs from these latter areas, but it is possible that they have lower stimulation thresholds due to differences in axon diameter or orientation.

### DBS influences local activity in the STN

DBS suppressed STN LFP power in the low beta frequency range, consistent with previous reports ( Kühn *et al.* , 2008 ; Bronte-Stewart *et al.* , 2009 ; Eusebio *et al.* , 2011 ; Whitmer *et al.* , 2012 ). Furthermore, the extent of low beta power suppression during DBS correlated with clinical motor improvement. Previously this had only been demonstrated immediately after cessation of DBS ( Kühn *et al.* , 2008 ). The suppression of low beta power in the STN by DBS is somewhat paradoxical given that the only cortical drive to be weakened by DBS was that from mesial premotor cortical areas which preferentially involved upper beta band activity. This might be taken to argue that the pattern of local oscillatory synchronization in the STN is determined by more than simply the nature of cortical drives, and allows for subcortical loops that amplify or generate activity in the beta, particularly low beta frequency band in the STN. The reciprocal connectivity between STN and globus pallidus externus is often cited in this regard ( Holgado *et al.* , 2010 ; Cruz *et al.* , 2011 ; Moran *et al.* , 2011 ; Tachibana *et al.* , 2011 ; Marreiros *et al.* , 2012 ). These subcortical loops promoting activity in the beta band may then also be weakened by DBS, an action shared by dopaminergic therapy ( Kühn *et al.* , 2006 *b* , 2009 ; Weinberger *et al.* , 2006 ; Ray *et al.* , 2008 ; Marreiros *et al.* , 2012 ).

### Conclusions and clinical implications

Experimental and computational modelling studies suggest that an abnormally strengthened hyperdirect pathway between motor cortical areas and the subthalamic nucleus is a prerequisite for pathological oscillations in basal ganglia circuits in Parkinson’s disease. The strength and changes in the strength of such local basal ganglia oscillations with dopaminergic therapy correlate with the extent of motor impairment and changes in motor impairment in this condition ( Kühn *et al.* , 2006 *b* , 2009 ; Weinberger *et al.* , 2006 ; Ray *et al.* , 2008 ; Chen *et al.* , 2010 ; López-Azcárate *et al.* , 2010 ; Özkurt *et al.* , 2011 ; Sharott *et al.* , 2014 ). Studies in rodent models of Parkinsonism have suggested that subthalamic DBS, another effective treatment of Parkinson’s disease, may at least partially act by disrupting the hyperdirect pathway through antidromic activation—thereby removing one of the necessary conditions for pathological oscillations ( Li *et al.* , 2007 , 2012 ; Dejean *et al.* , 2008 ; Gradinaru *et al.* , 2009 ; Holgado *et al.* , 2010 ). Using simultaneous MEG and LFP recordings we demonstrate that the therapeutic effects of DBS in parkinsonian patients are indeed associated with a selective attenuation of the presumed hyperdirect drive to the STN, and diminished oscillatory activity in this nucleus. The lack of and presence of clinical correlations with these respective effects adds further weight to the notion that a strengthened hyperdirect pathway is a prerequisite for locally generated or amplified beta activity but that it is the severity of the latter that may determine or index motor impairment ( Holgado *et al.* , 2010 ; Moran *et al.* , 2011 ; Marreiros *et al.* , 2012 ).

Our findings also reinforce the principle that different functional cortico-subcortical loops passing through the basal ganglia may not only be characterized by their differential cortical topography, but also by the predominant frequency range of the synchronized activity that they conduct ( Fogelson *et al.* , 2006 ; Hirschmann *et al.* , 2011 ; Litvak *et al.* , 2011 ). Thus temporal, lateral motor and mesial motor cortical loops could be distinguished by their relative preference for driving activities in the alpha, low beta and high beta ranges. A critical future challenge then is to define how activities in these different loops may underlie different symptoms. Thereafter stimulation could be further refined and made patient specific by tailoring both its delivery ( Bour *et al.* , 2015 ) and pattern ( Cagnan *et al.* , 2013 ; Little *et al.* , 2013a ) according to the nature of the LFP activities recorded at the stimulation target.

## Funding

This work was supported by the Medical Research Council (MR/K022172/1 to A.O and MC_UU_12024/1 to P.B) and the National Institute for Health Research University College London Hospitals and the National Institute for Health Research Oxford Biomedical Research Centre. The UK MEG community is supported by Medical Research Council grant MR/K005464/1. The Wellcome Trust Centre for Neuroimaging is supported by core funding from the Wellcome Trust 091593/Z/10/Z. The Unit of Functional Neurosurgery is supported by the Parkinson Appeal UK, and the Monument Trust.

## Supplementary material

Supplementary material is available at *Brain* online.

## Supplementary Material

Supplementary Data

## Abbreviations

DBS =: deep brain stimulation
DICS =: dynamic imaging of coherent sources
LFP =: local field potential
MEG =: magnetoencephalography
UPDRS =: Unified Parkinson’s Disease Rating Scale
SMA =: supplementary motor area
STN =: subthalamic nucleus
