# Supplementary material for: Deep brain stimulation modulates synchrony within spatially and spectrally distinct resting state networks in Parkinson’s disease
Source: Brain. 2016 Mar 26;139(5):1482–96. doi: 10.1093/brain/aww048 (PMC4845255; doi:10.1093/brain/aww048)
Supplement: Supplementary Data [file aww048_supplementary_data.zip › brain-2015-01903-File013.pdf]

Supplementary Figure 1. STN-MEG coherence topography is preserved despite artefact rejection. (a) 2D topography plot of all 275 MEG sensors, with the interpolated heat map representing the mean proportion of times each channel was rejected in our analysis across subjects. Value of 0 indicates that the channel was never rejected, whilst a value of 1 means that the channel was always rejected. Channels were rejected due to the presence of large amplitude jump artefacts in the MEG signal (see Supplementary Methods). Images for left STNs have been reflected through the midline for display purposes. Most frequently rejected channels overlie fronto-temporal regions ipsilateral to the stimulation. (b) Group mean of normalized DICS beamformer images in the no DBS condition with all MEG channels included for source space analysis. (c) Group mean of normalized DICS beamformer images in the no DBS condition with channels heavily contaminated with jump artefacts rejected prior to source space analysis. (d) Group mean of normalized DICS beamformer images during DBS with channels heavily contaminated with jump artefacts rejected prior to source space analysis. In (b), (c) and (d) unthresholded alpha (blue) and beta (yellow) coherence is superimposed onto a T1 weighted canonical MRI. Colour bar represents normalized coherence in arbitrary units. Values greater than 1 indicate that activity in that voxel is consistently greater than the mean across the image. Coronal, sagittal and axial sections through the image are displayed with cross-hairs centred on the image maxima for beta (upper panels) and alpha (lower panels).

Supplementary Figure 2. Spectral mixing and the estimation of phase delays from simulated data. Phase delays are estimated from the slope of the phase-frequency regression line for the high and low beta frequency ranges when (a) frequencies within these ranges are distinct – low beta 11-20 Hz and high beta 21-30 Hz and (b) frequencies within these ranges overlap by 1 Hz – low beta 11-21 Hz and high beta 21-30 Hz. The SNR and estimated delays

are shown in the title of each plot. The red and black regions of phase-frequency plot show the low and high beta frequency ranges, respectively whilst the thick red and black lines are linear regression fits over these ranges.

Supplementary Figure 3. Effects of 130 Hz DBS on cortical power. Group SPMs showing the effect of DBS on cortical power in alpha (a) and beta frequency ranges (b). Values indicated by the colour bar are t statistics. The SPMs are superimposed on a T1 weighted MRI with the cross-hairs centred on the locations of the coherence maxima for the alpha (a) and beta (b) bands. Positive t values indicate voxels where power off DBS was greater than power on DBS and vice versa. Cortical power in both bands was not found to be significantly influenced by DBS following correction for multiple comparisons using random field theory ($P < 0.05$ corrected, cluster forming threshold $P < 0.01$ uncorrected; the t tests had 77 degrees of freedom).
