# Supplementary material for: Deep brain stimulation modulates synchrony within spatially and spectrally distinct resting state networks in Parkinson’s disease
Source: Brain. 2016 Mar 26;139(5):1482–96. doi: 10.1093/brain/aww048 (PMC4845255; doi:10.1093/brain/aww048)
Supplement: Supplementary Data [file aww048_supplementary_data.zip › brain-2015-01903-File002.pdf]

Supplementary Material

Supplementary Methods

Patient and Surgical details

Patients were diagnosed with Parkinson's disease according to the Queen Square Brain Bank criteria (Gibb and Lees, 1988). All patients, except cases 10 and 11, displayed improvements in motor UPDRS part III motor scores during chronic DBS evaluated at 6 months post-surgery. Details of localisations of the individual DBS electrode contacts that were used for stimulation and LFP recording are provided in table 1. Recordings were approved by the National Research Ethics Service Committee South Central – Oxford B, and the patients gave written informed consent prior to participation.

The permanent DBS electrode implanted in the STN was model 3389 (Medtronic Neurological Division, Minneapolis, MN) with four platinum-iridium cylindrical surfaces (1.27 mm diameter and 1.5 mm length) and a centre-to-centre separation of 2 mm. The contacts were numbered 0 (lowermost, lying in the inferior portion of the STN) to 3 (uppermost, lying in the superior portion of the STN). Further details of the operative procedure can be found in (Foltynie and Hariz, 2010; Foltynie et al., 2011).

The locations of the electrodes were confirmed following implantation with immediate postoperative fast spin-echo T2-weighted magnetic resonance imaging (MRI) with a Leksell frame still in situ. Stainless steel electrode extension cables were externalized through the scalp to enable recordings prior to connection to a subcutaneous DBS pacemaker, implanted in a second operative procedure seven days later.

Data analysis

All analyses were performed using custom MATLAB scripts in combination with the SPM12 (http://www.fil.ion.ucl.ac.uk/spm/software/) (Litvak et al., 2011b), Data Analysis in Source Space (DAiSS, http://www.fil.ion.ucl.ac.uk/spm/ext/#DAiSS) and Fieldtrip (http://www.ru.nl/neuroimaging/fieldtrip/) toolboxes (Oostenveld et al., 2011).

Deleted: http://code.google.com/p/spm-beamforming-toolbox/

Only electrodes in which at least one of the recording pair of contacts (0-2) was located within the STN were used for analysis (Yoshida et al., 2010; see also Table 1). This led to the exclusion of data from three electrodes. Data from a further three electrodes were excluded as

Field Code Changed

the associated MEG signal contained significant artefacts, affecting greater than 150 channels during the 130 Hz DBS condition. The cut off of 150 bad channels was chosen empirically based on analysis presented below where we compare source reconstruction accuracy in the no DBS condition before and after channel rejection. This left 24 subthalamic nuclei for further analysis.

Deleted: (see below)

Data pre-processing and sensor level analysis

In previous work, we have described our approach for obtaining an accurate estimate of head location for each recording run, based on both an initial measurement of head position and continuous tracking of HPI coils (Litvak et al., 2012, Oswal et al., 2015). In practice we found that both the initial measurement and parts of the continuous head tracking could be disrupted by the presence of ferromagnetic wires and the DBS pulses – therefore our approach had to be robust to such interferences. The full details of our approach are defined in the aforementioned studies, but the overall idea is based on the fact that if head position is accurately tracked, the pairwise distances between the HPI coils should stay constant both within a recording run and between recording runs in the same scanning session. Time points where head tracking was lost within a recording run could then be identified and corrected by interpolation, such that the average of the resulting interpolated values would yield a robust estimate of head position. Head and sensor locations were always visually inspected for each subject and compared across runs to make sure that there were no gross outliers or misregistrations.

The raw data files from the CTF-MEG and BrainVision systems were converted to SPM MATLAB-based format. The LFP data were interpolated to 2400Hz, matching the sampling rate of the MEG data. MEG and LFP recordings were then aligned using the synchronisation signal and combined (Oswal et al., 2015).

Visual inspection of the individual MEG channel data obtained during monopolar DBS at 130 Hz revealed high amplitude jumps in some of the channels that lasted only a few samples (Oswal et al., 2015). Prior to further analysis, we rejected channels where the number of jumps exceeded 1000 - where we defined a jump as an absolute difference in the magnetic field between adjacent samples of greater than 10^5 fT (femto Tesla). This procedure removed channels that were worst affected by jumps. These channels were removed for the DBS and

no DBS conditions for each subject in order to ensure that the data were comparable. In separate analysis we ensured that beamformer localisation of source activity was not significantly influenced by our channel rejection procedure (see Supplementary Results).

In the remaining channels with jumps, we repaired the jumps, using an interpolation-based approach. Following the detection of a jump, a fixed segment of data (120ms) either side of the jump was examined and DBS stimulation pulse peaks within it were identified. The data between DBS pulse peaks greater than two pulses away from the jump on either side were averaged to produce a signal for interpolating the region contaminated with the jump. The contaminated region of data was defined as the segment of data between two DBS pulse peaks of the jump on either side. Finally, the mean of the post-jump data segment was adjusted to correspond to that of the pre-jump data, ensuring smooth fixing of the jump (Oswal et al., 2015). Following jump correction, the data were downsampled to 300 Hz and high pass filtered above 1Hz prior. Margins of 20s were then removed from the beginning and the end of the no DBS and 130 Hz DBS runs.

In addition to jump artefacts, we observed additional artefacts related to: 1) the movement of ferromagnetic electrode extension cables following arterial pulsations timed to each heartbeat and 2) individual stimulation pulses occurring at the stimulation frequency and its harmonics. We have shown that coherence can be accurately recovered using beamforming despite the presence of such artefacts (Oswal et al., 2015).

Generation of DICS beamformer images

In order to limit the effects of volume conduction we defined the beamformer source orientations as the normalised imaginary part of the cross-spectral density vector between the recorded LFP and the x, y and z orientations of the MEG source (Nolte et al., 2004; Litvak et al., 2010). The resulting coherence values were then linearly interpolated to produce 3D volumetric images with 2 mm resolution for visualisation. Common spatial filters were used to generate images for the DBS and no DBS conditions. Images were smoothed with an 8 mm isotropic Gaussian kernel to ensure conformance to the assumptions of random field theory prior to statistical analysis in SPM.

Beamformer time series extraction

For source extraction from pre-defined regions of interest (M1 and image peaks) we used our previously published methods (Oswal et al., 2014). The idea is that in order to minimise the influence of potentially artefactual contributions from outside a predefined ROI, it is possible to project the data to a subspace spanned by the leadfields within an ROI. We used a 1cm cubic ROI centred on the location of the peak, with leadfield sampling at 5mm spaced intervals on the cubic region. The covariance matrix for LCMV beamforming may then be computed after this linear projection step. In this particular analysis we employed a more principled way for determining the optimum amount of regularisation for the data covariance by using Bayesian principal component analysis (see Oswal et al., 2014 for further details of the potential advantages of this approach). Once again common filters were used for the two experimental conditions, and source orientations were defined as the normalised imaginary part of the cross-spectral density vector between the recorded LFP and the x, y and z orientations of the MEG source.

Directionality of STN-cortical coupling

The approach we use for generating surrogate data has been shown to suppress weak asymmetries that are not due to genuine time lagged interactions (Haufe et al., 2013). Therefore, taking the example of two signals A and B with A Granger causing B, the Granger causality from A to B should be higher for the original than for the time reversed data. In contrast however, the estimate of causality from B to A, should be increased by time reversal.

Having established the effective directionality of functional coupling, we estimated the phase delays between the STN activities demonstrating peaks in coherence and cortical regions. In order to do this we assumed that any time delay was fixed over peak frequencies. Accordingly, the time delay, $\Delta T,$ between the regions is given by $\Delta T = abs(\emptyset_{cortex} - \emptyset_{STN})/2\pi f$ where f indicates the frequency of the signals and $abs(\emptyset_{cortex} - \emptyset_{STN})$ represents the magnitude of the phase delay between signals (see Fogelson et al., 2006 for similar use of this approach in estimating time delays). Phase differences were estimated from complex cross-spectra after robust averaging (see above). We distinguished two frequency ranges, 13-21 Hz and 21-30 Hz within our data (see results section) and regressed phase differences against frequency in order to approximate net delays. Only data points for

which there was a significant linear relationship between phase and frequency ($p < 0.05$) were included in the computation of delays.

Deleted: ¶

Formatted: Font: Bold

Supplementary Results

Defining resting state networks in PD and handling DBS related artefacts

A key goal of our analysis was to determine the effects of DBS on resting state networks involving the stimulation target, the STN. One of these can be tracked by LFP recordings alone in the form of the coherence between the two STNs. Spectra of the coherence between bipolar contacts 0-2 of the stimulated and contralateral STNs are presented in panel d of Figure 1. Peaks in resting coherence (no DBS condition) occur at similar frequencies as peaks in the resting spectral power profiles.

A further two resting state networks can be identified through simultaneous MEG and LFP recordings by characterising STN – cortical coupling in the alpha (7-12 Hz) and beta (13-30 Hz) frequency bands (Hirschmann et al., 2011; Litvak et al., 2011a). First we determined if these same two networks could be identified in the current cohort in the no stimulation condition. Panel b of Supplementary Figure 1 shows mean images of normalised STN-cortical coherence for all included STNs separately for the alpha and broad beta bands. Note that all images for left STNs have been reflected across the median sagittal plane such that the resulting networks appear on the right hemisphere. The present results match very closely those previously reported in that two spatially and spectrally segregated STN-cortical networks exist. A broad beta band network exists between the STN and supplementary motor and premotor regions (peak at MNI co-ordinates 18 -14 68 corresponding to right superior frontal gyrus), while a separate alpha band network exists between STN and temporal areas (peak at MNI co-ordinates 48 -34 18 corresponding to the right superior temporal gyrus).

Deleted: 48 -34 18

Deleted: 18 -14 68

Prior to exploring how these two networks respond to STN DBS we checked that we could still identify the two networks after rejecting MEG channels that were contaminated with artefacts. In our analysis of the effects of DBS we rejected hemispheres where over 150 channels were contaminated with jumps in the corresponding MEG signal. For the remaining hemispheres, Supplementary Figure 1a shows a 2D topographic plot of all 275 channels, with the interpolated heat map representing the mean proportion of times each channel was

rejected in our analysis across subjects. A value of 0 indicates that the channel was never rejected, whilst a value of 1 means that the channel was always rejected. The images for left STNs have been reflected through the midline for display purposes. It is evident that the most frequently rejected channels overlie fronto-temporal regions, where the monopolar stimulation current flows on its path between the implanted stimulated contact and the cutaneous anodal electrode on the chest wall. Supplementary Figure 1c shows the new source reconstructions of the alpha and broad beta networks in the no DBS condition, following channel rejection. Supplementary Figure 1d shows source reconstructions of the alpha and beta networks during DBS, following channel rejection. The good concordance between Supplementary Figures 1b and 1c highlights that it is possible to accurately recover the resting alpha and beta networks in our patients despite rejecting severely contaminated channels.

Testing for wash-out and delayed onset effects of DBS on STN-cortical coherence

In subsidiary analysis we sought to assess for possible wash out and delayed onset effects of DBS by comparing STN-cortical beta coherence in the first and second halves of each recording for both stimulation conditions. This analysis was performed separately for LCMV beamformer extracted source timeseries from the STN – mesial premotor and the STN – lateral motor networks. Reassuringly, for both networks and both stimulation conditions, no significant differences in split half beta coherence were observed (Maximum t value, t_{22} = 1.7, corresponding to $p > 0.1$).

Spectral mixing and the estimation of phase delays

Our estimated phase delay for coupling in the high beta band between cortex and the STN was indeed longer than what one may expect for monosynaptic conduction. Nevertheless an important technical consideration which we have alluded to in the discussion is that there is likely to be some degree of spectral (and spatial) overlap of the activities supported by the indirect and hyperdirect pathways (Cassidy and Brown, 2003) .

In order to provide an empirical validation of the effect of such frequency mixing, we generated a time series, *X*, consisting of low (13-20 Hz) and high beta activity (21-30 Hz) by including a sinusoid at each integer frequency within both sub-bands. A second time series, *Y*, was generated in the same way but a phase lag was added to each constituent frequency

Deleted: ¶

Formatted: Font: Bold, Underline

Formatted: Font: Bold, Underline

Formatted: Font: Bold, Underline

Formatted: Subscript

Deleted: 3

Formatted: Subscript

such that there was a fixed delay between *X* and *Y* over the low and high beta frequency ranges. The delay over the low beta frequency range was selected to be 40ms (mimicking our estimated cortico-STN phase delay for this frequency range) while the delay over the high beta frequency range was selected to be 10ms. Random Gaussian noise was added to each time series and phase delays were estimated using our regression approach at three different SNR levels, ranging from 0.01 to 1. In an additional simulation the high and low beta frequency ranges were overlapped by just 1 Hz before generating the two time series such that low beta was defined over the range 13-21 Hz and high beta was defined over the range 21-30 Hz. The purpose of this overlap was to simulate (minimal) spectral mixing.

The results of this simulation are now shown in Supplementary Figure 2. The individual plots display phase-frequency profiles with low and high beta frequencies indicated by the regions in red and black respectively. The thick red and black lines are linear regression lines for low (13-20 Hz) and high (21-30 Hz) beta frequencies which are used to estimate phase delays. Panel a displays the case in which the low and high beta frequency ranges are spectrally distinct, whereas panel b displays the case in which an overlap of 1 Hz exists. In the case where the high and low beta frequency ranges are distinct phase delays are estimated remarkably well across the range of SNR levels. When a minimal and abrupt 1 Hz overlap existed however, estimation of the slope of the regression line was biased such that estimated phase delays were prolonged. In this case, delays in the conduction of low beta frequencies were estimated at ~48ms (true value was 40ms) whilst delays in the conduction of high beta frequencies were estimated at ~20ms (true value was 10ms). In reality, frequency mixing may occur over a wider band of frequencies and may not be so abrupt in its onset as in our simple simulation.

Although our estimated delays may not correspond exactly to delays within the hyperdirect and indirect pathways, our results indicate that net delays in the direction of cortex to STN are much shorter in the high than in the low beta frequency range – consistent with the notion that activities in these two bands are more subserved by the hyperdirect and indirect pathways respectively.

Formatted: Font: (Intl) Arial Unicode MS, English (U.K.)

Supplementary Table 1.

Experimental condition	Mesial premotor No DBS	Mesial premotor 130 Hz DBS	Lateral No DBS	Lateral 130 Hz DBS
Low beta sub-band	0.031 (0.002)	0.023(0.003)	0.030(0.003)	0.028(0.003)
High beta sub-band	0.042 (0.004)	0.032(0.004)	0.029(0.003)	0.030(0.002)

Supplementary Table 1. Coherence values with standard errors in brackets are provided for coupling in the low and high beta sub-bands between the STN and cortical regions without and with DBS (see Results).

Supplementary References

Cassidy M, Brown P (2003) Spectral phase estimates in the setting of multidirectional coupling. J Neurosci Methods 127:95–103.

Foltynie T, Hariz MI (2010) Surgical management of Parkinson's disease. Expert Rev Neurother 10:903–914.

Foltynie T, Zrinzo L, Martinez-Torres I, Tripoliti E, Petersen E, Holl E, Aviles-Olmos I, Jahanshahi M, Hariz M, Limousin P (2011) MRI-guided STN DBS in Parkinson's disease without microelectrode recording: efficacy and safety. J Neurol Neurosurg Psychiatry 82:358–363.

Haufe S, Nikulin V V, Müller K-R, Nolte G (2013) A critical assessment of connectivity measures for EEG data: a simulation study. Neuroimage 64:120–133.

Hirschmann J, Özkurt TE, Butz M, Homburger M, Elben S, Hartmann CJ, Vesper J, Wojtecki L, Schnitzler A (2011) Distinct oscillatory STN-cortical loops revealed by simultaneous MEG and local field potential recordings in patients with Parkinson's disease. Neuroimage 55:1159–1168.

Litvak V, Eusebio A, Jha A, Oostenveld R, Barnes GR, Penny WD, Zrinzo L, Hariz MI, Limousin P, Friston KJ, Brown P (2010) Optimized beamforming for simultaneous MEG and intracranial local field potential recordings in deep brain stimulation patients. Neuroimage 50:1578–1588.

Litvak V, Jha A, Eusebio A, Oostenveld R, Foltynie T, Limousin P, Zrinzo L, Hariz MI, Friston K, Brown P (2011a) Resting oscillatory cortico-subthalamic connectivity in patients with Parkinson's disease. Brain 134:359–374.

Litvak V, Mattout J, Kiebel S, Phillips C, Henson R, Kilner J, Barnes G, Oostenveld R, Daunizeau J, Flandin G, Penny W, Friston K (2011b) EEG and MEG data analysis in SPM8. Comput Intell Neurosci 2011:852961.

Nolte G, Bai O, Wheaton L, Mari Z, Vorbach S, Hallett M (2004) Identifying true brain interaction from EEG data using the imaginary part of coherency. Clin Neurophysiol 115:2292–2307.

Oostenveld R, Fries P, Maris E, Schoffelen J-M (2011) FieldTrip: Open source software for advanced analysis of MEG, EEG, and invasive electrophysiological data. Comput Intell Neurosci 2011:156869.

Oswal A, Jha A, Neal S, Reid A, Bradbury D, Aston P, Limousin P, Foltynie T, Zrinzo L, Brown P, Litvak V (2015) Analysis of simultaneous MEG and intracranial LFP recordings during Deep Brain Stimulation: a protocol and experimental validation. J Neurosci Methods Available at: http://www.sciencedirect.com/science/article/pii/S016502701500432X.

Oswal A, Litvak V, Brown P, Woolrich M, Barnes G (2014) Optimising beamformer regions of interest analysis. Neuroimage 102 Pt 2:945–954.

Yoshida F, Martinez-Torres I, Pogosyan A, Holl E, Petersen E, Chen CC, Foltynie T, Limousin P, Zrinzo LU, Hariz MI, Brown P (2010) Value of subthalamic nucleus local field potentials recordings in predicting stimulation parameters for deep brain stimulation in Parkinson’s disease. J Neurol Neurosurg Psychiatry 81:885–889.
