# Supplementary figures and images for: Deep brain stimulation modulates synchrony within spatially and spectrally distinct resting state networks in Parkinson’s disease
Source: Brain. 2016 Mar 26;139(5):1482–96. doi: 10.1093/brain/aww048 (PMC4845255; doi:10.1093/brain/aww048)

Supplementary Figure 1

a

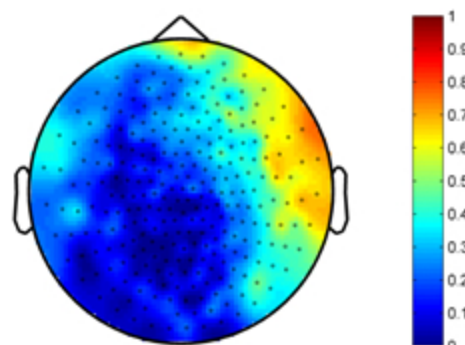

Beta

b

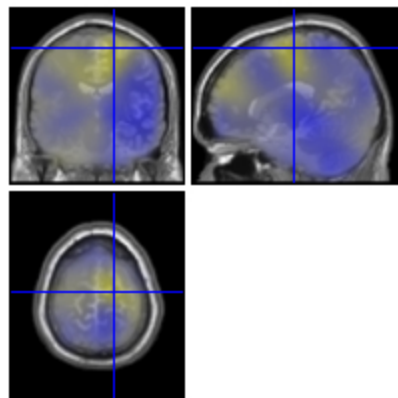

c

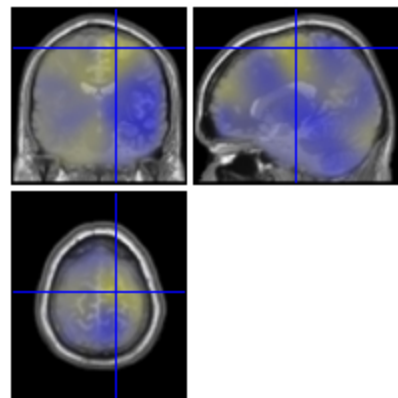

d

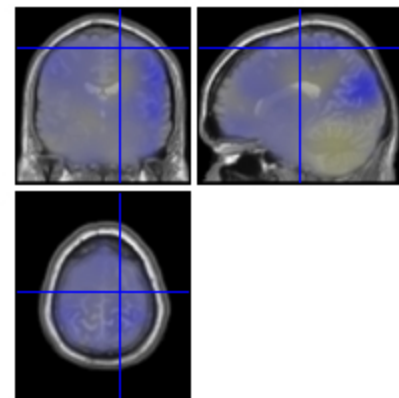

Alpha

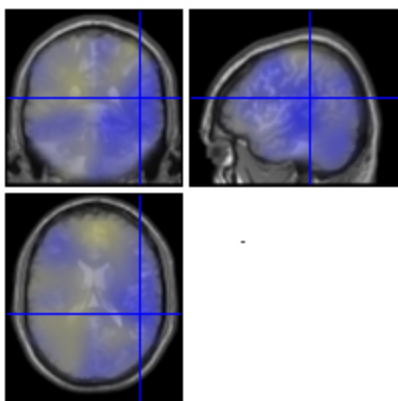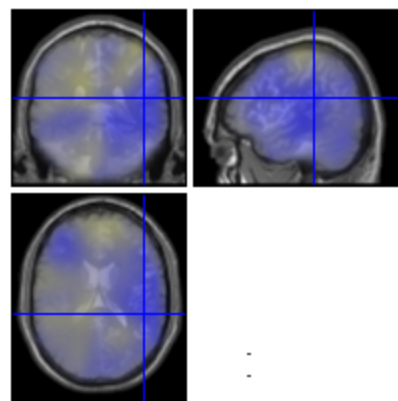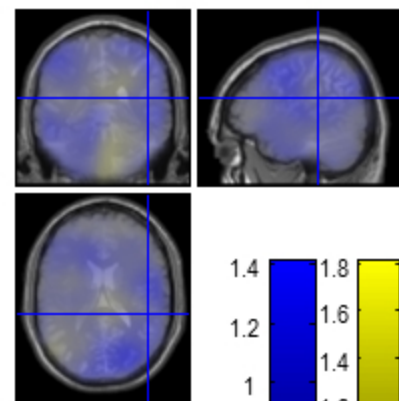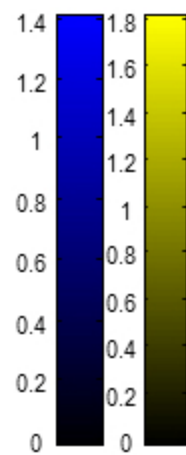

Supplement: Supplementary Data [file aww048_supplementary_data.zip › brain-2015-01903-File009.pdf]

## Supplementary Figure 2

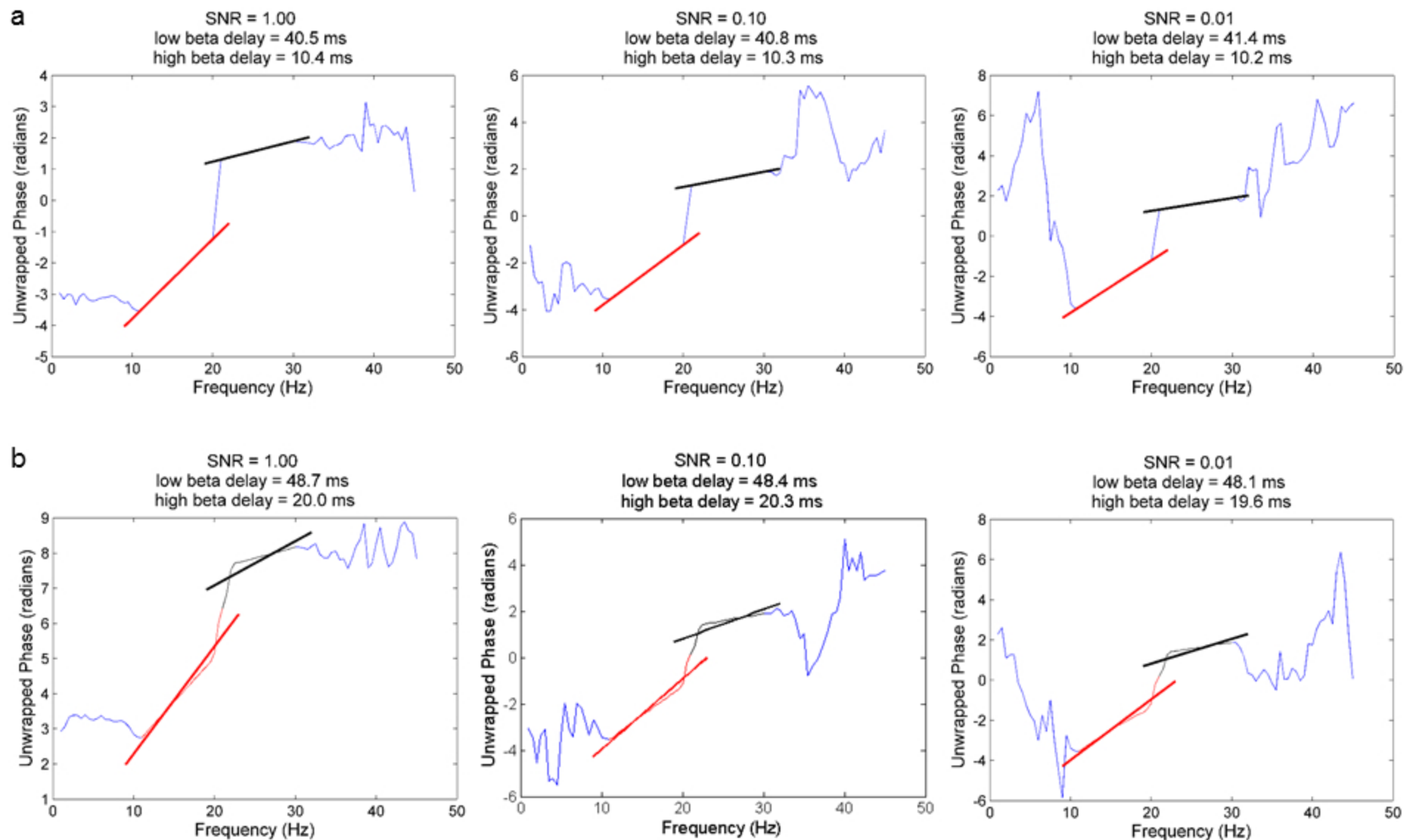

Supplement: Supplementary Data [file aww048_supplementary_data.zip › brain-2015-01903-File010.pdf]

Supplementary Figure 3

a

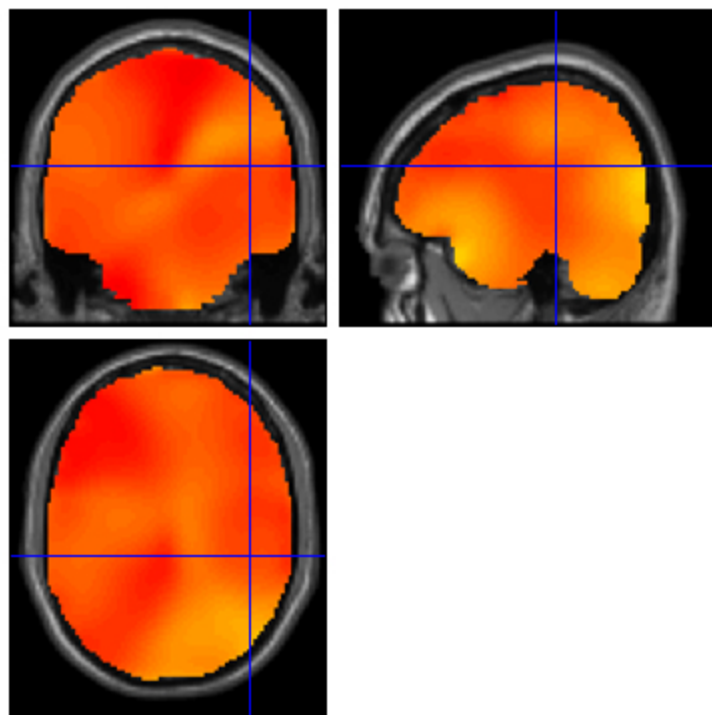

b

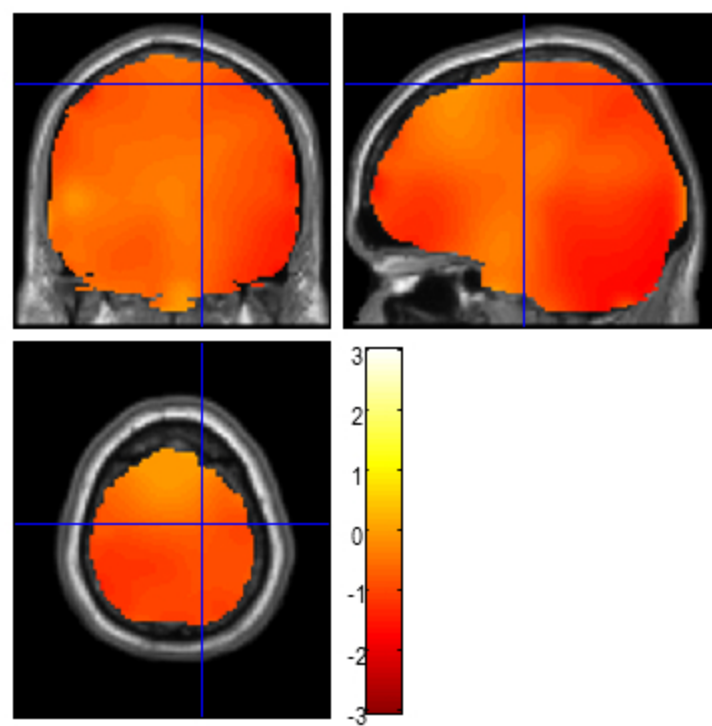

Supplement: Supplementary Data [file aww048_supplementary_data.zip › brain-2015-01903-File011.pdf]

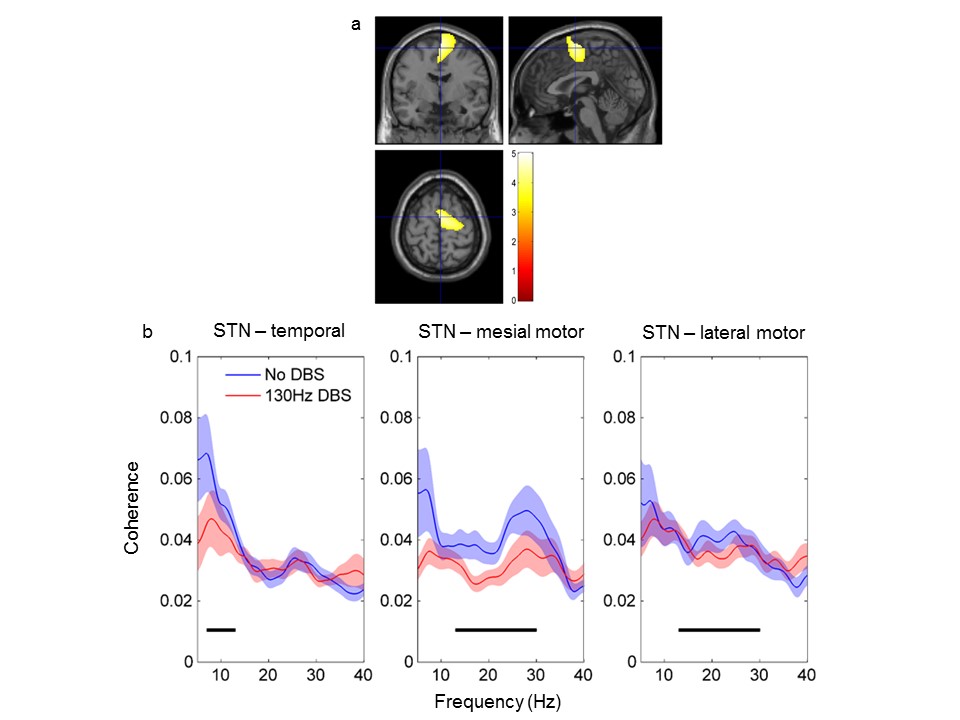

Supplement: Supplementary Data [file aww048_supplementary_data.zip › brain-2015-01903-File012.jpg]
